# MHC class II restricted neoantigen peptides predicted by clonal mutation analysis in lung adenocarcinoma patients: implications on prognostic immunological biomarker and vaccine design

**DOI:** 10.1186/s12864-018-4958-5

**Published:** 2018-08-03

**Authors:** Weijing Cai, Dapeng Zhou, Weibo Wu, Wen Ling Tan, Jiaqian Wang, Caicun Zhou, Yanyan Lou

**Affiliations:** 1 Shanghai Pulmonary Hospital affiliated with Tongji University School of Medicine, Shanghai, 200092 China; 2 YuceBio Technology Co., Ltd, Shanghai, 201203 China; 3 Division of Hematology and Oncology, Mayo Clinic, Jacksonville, FL 32224 USA

**Keywords:** Lung cancer, Neo-antigen, Cancer vaccine, PD1 checkpoint blocking antibody

## Abstract

**Background:**

Mutant peptides presented by MHC (major histocompatibility complex) Class II in cancer are important targets for cancer immunotherapy. Both animal studies and clinical trials in cancer patients showed that CD4 T cells specific to tumor-derived mutant peptides are essential for the efficacy of immune checkpoint blockade therapy by PD1 antibody.

**Results:**

In this study, we analyzed the next generation sequencing data of 147 lung adenocarcinoma patients from The Cancer Genome Atlas and predicted neoantigens presented by MHC Class I and Class II molecules. We found 18,175 expressed clonal somatic mutations, with an average of 124 per patient. The presentation of mutant peptides by an HLA(human leukocyte antigen) Class II molecule, HLA DRB1, were predicted by NetMHCIIpan3.1. 8804 neo-peptides, including 375 strong binders and 8429 weak binders were found. For HLA DRB1*01:01, 54 strong binders and 896 weak binders were found. The most commonly mutated genes with predicted neo-antigens are *KRAS, TTN, RYR2, MUC16, TP53, USH2A, ZFHX4, KEAP1, STK11, FAT3, NAV3* and *EGFR*.

**Conclusions:**

Our results support the feasibility of discovering individualized HLA Class II presented mutant peptides as candidates for immunodiagnosis and immunotherapy of lung adenocarcinoma.

**Electronic supplementary material:**

The online version of this article (10.1186/s12864-018-4958-5) contains supplementary material, which is available to authorized users.

## Background

The efficacy of therapeutic effect of immune checkpoint blockade such as PD1 and CLTA4 antibodies is hypothesized to be dependent on mutant peptide epitopes which cause the T cell dependent cytotoxicity toward tumor cells. Epitopes for CD4 T cells are proposed to be a major mechanism. In mouse models, both artificial protein antigens and mutant peptide antigens derived from tumor cells were found to elicit tumorcidal T cell responses [1–3]. Clinical trials using long peptides or mRNA to deliver CD4 T cell epitopes to dendritic cells have shown success in inducing mutant peptide-specific CD4 T cells and their association with anti-tumor efficacy [4–6].

In this study, we analyzed next generation sequencing data from 147 lung adenocarcinoma patients deposited in the Cancer Genome Atlas, to identify both the driver and passenger mutations which may be presented by HLA Class II molecules. Due to the complexity of polymorphisms of both alpha and beta chains of HLA Class II molecules, we only studied the binding of mutant peptides to HLA DRB1 molecules that pair with an invariant alpha chain, HLA DRA.

## Methods

### Standardization and tracking of mutation data from TCGA

We collected mutations of lung adenocarcinoma from TCGA [7]. The data collection criteria was established as follows: 1, Tumor and matched normal adjacent tissue were included; 2, Samples that contain all somatic mutation, expression, SNP (single nucleotide polymorphism) array information were included; 3, Tumor samples from same patients were removed; 4, Samples with purity lower than 20% or ploidy larger than 6 were removed, purity and ploidy were reported by AbsCN-seq [8].

To remove common sequencing artifacts or residual germ line variation, each mutation was subjected to a ‘Panel of Normals’ filtering process using a panel of over 600 BAM files from normal samples. Mutations observed more than 1% in the panel of normals, dbSNP [9] or 1000G [10] were removed. Finally, all mutations with covered reads less than 10X were filtered out.

### Purity and ploidy analysis

Purity and ploidy were estimated by AbsCN-seq, a software developed for WES (whole exon sequencing) data, based on SNV (single nucleotide variations) frequency and segment copy number.

### Mutation clonality analysis

After estimating the tumor purity, we calculated the CCF (cancer cell fraction) for each mutation. The CCF is the percentage of tumor cells harboring a given mutation. Clonal mutations have a true CCF of 1, and subclonal mutations have a true CCF < 1. The observed allele counts correspond to a probability density of the CCF, which can be estimated with the following equation, where q(*m*) is the local copy number at the given mutation *m*, a is purity, and CCF ranges from 0 to 1. pdf is probability density function, alt is the alternate allele counts, ref. is the reference allele counts [11].

$$ \mathrm{pdf}\left(\mathrm{CCF},\mathrm{m}\right)=\upbeta \mathrm{pdf}\left\{{\mathrm{CCF}}^{\ast}\upalpha, \mathrm{alt}\left(\mathrm{m}\right)/\left[{2}^{\ast}\left(1-\upalpha \right)\kern0.37em +\kern0.37em {\upalpha}^{\ast}\mathrm{q}\left(\mathrm{m}\right)\right]\kern0.37em +\kern0.37em 1,\mathrm{ref}\left(\mathrm{m}\right)+1\right\} $$

### Neo-peptides prediction

We first confirmed that the mutated genes were expressed by RNA-seq data. Genes with 3 or more reads covered were defined as expressed according to Kandoth et al. [12]. 29-mer polypeptides centered on mutated residues were scanned to identify candidate peptides binding to MHC Class I or II molecules [13], i.e., peptide sequences surrounding mutated amino acids resulting from missense mutations, frame-shift or non-frame-shift indels. The affinity of 8–11 peptides binding to MHC Class I molecules were predicted using the NetMHCPan2.4 binding algorithm [14]. The affinity of 15 mer peptides binding to MHC Class II molecules were predicted using the NetMHCIIPan3.1 binding algorithm [15]. Threshold for strong binding peptides is defined as half-maximum inhibitory concentration (IC50) < 50 nM; Threshold for weak binding peptides is defined as IC50 < 500 nM [15–17].

MHC Class II molecules include HLA DP, DQ, and DR molecules. These molecules are composed of alpha and beta subunits. For DP and DQ molecules, both alpha and beta subunits are polymorphic. DR molecules are composed by a polymorphic beta subunit and an invariant alpha subunit. In this study, we focused on HLA DRB1, the most prevalent beta subunit of HLA DR [18]. The frequencies of other DRB molecules (DRB3, 4 and 5) are 5 to 10 fold lower than DRB1 (reference [18]). Clearly DRB1 molecules are significantly more frequent in presenting neo-antigens.

## Results

To ensure high quality mutation calls for lung adenocarcinoma, stringent filters (Methods) were applied in sample and mutation collecting. A total of 40,229 somatic mutations in 147 lung adenocarcinomas were included for downstream analysis, including 26,296 missense, 8965 silent, 2061 nonsense, 911 splice site, 98 non-stop/read through, 1735 frame shift insertions/deletions (indels) and 163 inframe indels.

**Fig. 1:**
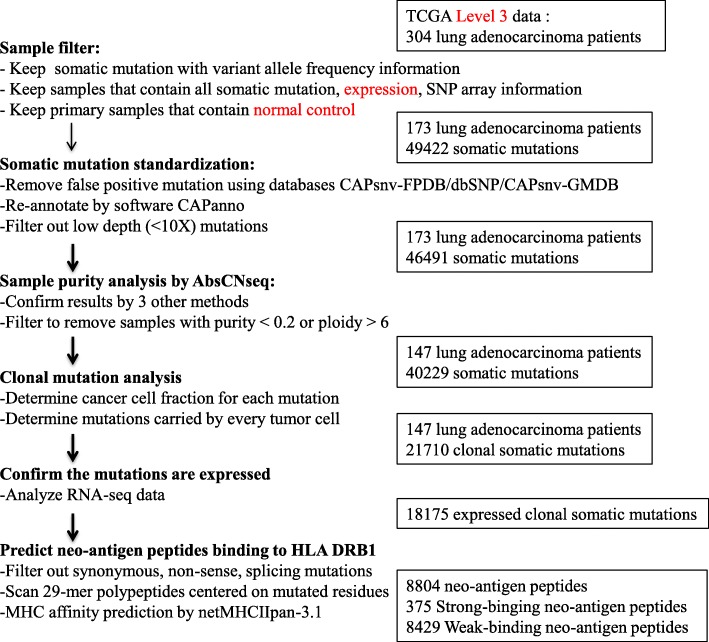
Flow chart of clonal mutation analysis and HLA-binding neo-antigen prediction for lung adenocarcinoma patients

We assessed the CCF(cancer cell fraction) of each mutation as described in Carter et al. [19] to assess whether mutations are clonal (i.e., present in all cancer cells). Mutations are considered clonal if the CCF is close to 1. To determine the CCF, we calculated the sample purity (i.e., the percentage of tumor cells in sample), ploidy (i.e., a measure of the number of chromosomes in a cell) and absolute copy number by Abs-CNseq. We further identified clonal mutations based on beta distribution. In total, we identified 21,710 clonal mutations (Fig. 1), including the known proliferation-related genes (e.g., TP53, KRAS, EGFR).

**Fig. 2:**
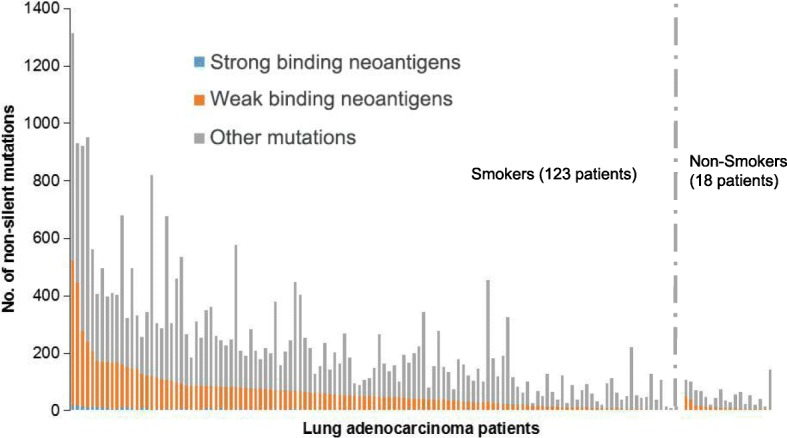
Predicted HLA-DRB1-binding neo-antigen mutant peptides in 147 lung adenocarcinoma patients. Somatic mutations were predicted by NetMHCIIPan3.1. All patients were lined up according to numbers of HLA-DRB1-binding neo-antigen mutations, including both strong-binders (SB, blue color) and weak-binders (WB, red color). Gray color indicates other mutations which do not bind to MHC Class II molecules. Smokers and non-smokers were analyzed separately

**Fig. 3:**
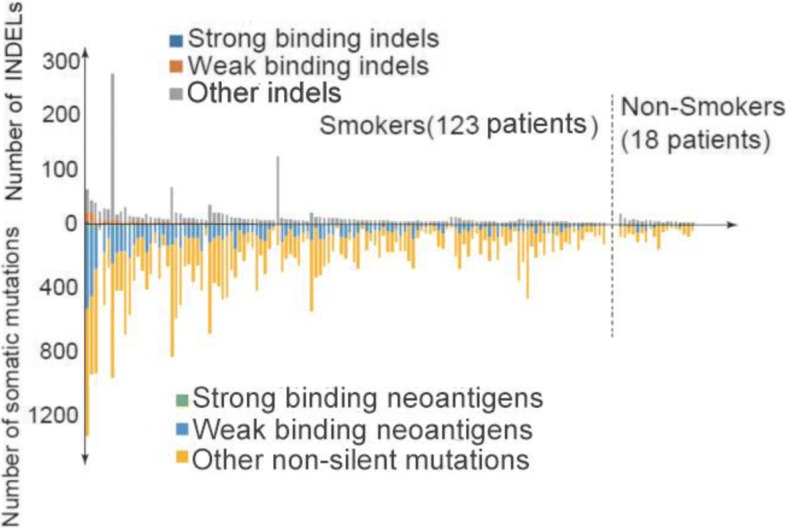
Predicted HLA-DRB1-binding INDEL mutant peptides in 147 lung adenocarcinoma patients

High-affinity candidate T cell epitopes were identified in silico by scanning of the mutant peptides resulting from missense mutations, frame-shift or non-frame-shift indels. T cell epitopes presented by MHC Class I molecules were predicted by NetMHCPan2.4 binding algorithm (Additional file 1: Table S1, Additional file 2: Table S2 and Additional file 3: Table S3). T cell epitopes presented by MHC Class II molecules were predicted by NetMHCIIPan3.1 binding algorithm. We focused on HLA DRB1, the most prevalent beta subunit of HLA DR which pairs with invariant alpha subunit HLA DRA [18]. In total, 8804 neo-peptides, including 375 strong binders and 8429 weak binders were found (Fig. 2). For DRB1*01:01, 950 neo-peptides, including 54 strong binders and 896 weak binders were found. The most commonly mutated genes with predicted neo-antigens are KRAS, TTN, RYR2, MUC16, TP53, USH2A, ZFHX4, KEAP1, STK11, FAT3, NAV3 and EGFR (Table 1). The exact mutated sequences are listed in Additional file 4: Table S4. The frequency of neo-peptides varies widely in individual patients of lung adenocarcinomas, from 0 to 523 (Fig. 2). Table 2 shows the distribution of neo-antigens in different HLA DRB1 alleles. DRB1*01:02, DRB1*12:01, DRB1*11:04, DRB1*01:01 were found to be the most frequent DRB1 alleles which present neo-antigens. High frequency of neo-peptides were found in hotspots of KRAS (Table 3, G12C or G12 V). INDEL mutations were found in most patients (Fig. 3). However, no linear correlation was found between SNV and INDEL mutations.

**Table 1:** Top mutated genes with predicted HLA DRB1 binding neo-peptides in lung adenocarcinoma patients in this study

Gene	No. of strong-binding neo-antigens	No. of weak-binding neo-antigens	Other mutant peptides	Total mutated peptides	Frequency of neo-antigens in 147 samples
KRAS	0	48	3	51	32.65%
TTN	1	44	101	146	22.45%
RYR2	0	36	43	79	20.41%
MUC16	2	35	58	95	20.41%
TP53	1	25	37	63	17.01%
USH2A	1	24	25	50	13.61%
ZFHX4	1	21	42	64	14.29%
KEAP1	1	19	10	30	13.61%
STK11	1	17	17	35	11.56%
FAT3	0	15	14	29	7.48%
NAV3	2	14	16	32	10.20%
EGFR	0	14	10	24	8.16%
SPTA1	0	13	31	44	8.84%
ANK2	0	13	14	27	7.48%
ADAMTS12	0	13	22	35	6.12%
PXDNL	0	12	11	23	8.16%
DMD	0	12	14	26	8.16%
ASPM	0	12	6	18	8.16%
LPHN3	1	11	10	22	8.16%
DNAH9	0	11	15	26	6.12%

**Table 2:** Number of predicted neo-antigen peptides presented by MHC Class II molecule HLA DRB1

HLA allele	No. of strong-binding neo-antigens	No. of weak-binding neo-antigens	Total neo-antigens	HLA frequency in Caucasian population	*P* value	Q value
DRB1*01:02	88	1174	1262	1.40%	1.57E-24	4.86E-23
DRB1*12:01	98	1046	1144	1.60%	9.03E-24	1.40E-22
DRB1*11:04	48	756	804	2.90%	4.04E-23	4.18E-22
DRB1*01:01	54	896	950	8.60%	1.93E-22	1.50E-21
DRB1*01:03	4	392	396	1.20%	1.11E-06	6.86E-06
DRB1*13:03	3	385	388	1.10%	6.80E-05	3.51E-04
DRB1*16:02	22	293	315	0.15%	2.52E-01	1.00E + 00
DRB1*03:01	4	303	307	12.20%	4.31E-01	1.00E + 00
DRB1*11:03	2	248	250	0.61%	9.68E-01	1.00E + 00
DRB1*08:03	3	225	228	0.24%	1.00E + 00	1.00E + 00
DRB1*07:01	6	217	223	13.40%	1.00E + 00	1.00E + 00
DRB1*04:05	3	217	220	0.67%	1.00E + 00	1.00E + 00
DRB1*04:01	2	213	215	8.80%	1.00E + 00	1.00E + 00
DRB1*08:04	7	190	197	0.20%	1.00E + 00	1.00E + 00
DRB1*10:01	12	179	191	0.85%	1.00E + 00	1.00E + 00
DRB1*09:01	4	175	179	1.00%	1.00E + 00	1.00E + 00
DRB1*04:04	0	151	151	3.90%	1.00E + 00	1.00E + 00
DRB1*13:05	4	137	141	0.25%	1.00E + 00	1.00E + 00
DRB1*13:02	0	137	137	4.90%	1.00E + 00	1.00E + 00
DRB1*16:01	1	124	125	1.40%	1.00E + 00	1.00E + 00
DRB1*08:01	2	121	123	2.30%	1.00E + 00	1.00E + 00
DRB1*11:01	4	116	120	5.60%	1.00E + 00	1.00E + 00
DRB1*13:01	0	116	116	5.60%	1.00E + 00	1.00E + 00
DRB1*11:02	0	113	113	0.28%	1.00E + 00	1.00E + 00
DRB1*04:08	0	107	107	0.39%	1.00E + 00	1.00E + 00
DRB1*15:01	2	103	105	13.50%	1.00E + 00	1.00E + 00
DRB1*14:01	1	88	89	2.60%	1.00E + 00	1.00E + 00
DRB1*15:02	0	55	55	0.72%	1.00E + 00	1.00E + 00
DRB1*04:07	0	54	54	1.10%	1.00E + 00	1.00E + 00
DRB1*04:03	1	49	50	0.79%	1.00E + 00	1.00E + 00
DRB1*04:02	0	49	49	1.10%	1.00E + 00	1.00E + 00

MHCII molecules which are significantly more frequent in presenting neo-antigens were labelled as bold according to *P* values. Significant levels were calculated using one sided Mann-Whitney U test

**Table 3:** Predicted HLA DRB1-binding neo-peptides of KRAS, EGFR, TP53, and MUC16 in lung adenocarcinoma patients in this study

Gene	Mutation	HLA	MHC affinity score(nM)	Neo-peptide	Frequency of neoantigens in 147 samples
*KRAS*	p.G12C	DRB1*01:01	214.21	VGACGVGKSALTIQL	14.97%
	p.G12 V	DRB1*01:02	81.75	VVGAVGVGKSALTIQ	10.20%
	p.G12A	DRB1*12:01	220.77	KLVVVGAAGVGKSAL	2.72%
	p.G12D	DRB1*11:03	280.09	KLVVVGADGVGKSAL	0.68%
	p.G12F	DRB1*08:04	89.1	LVVVGAWRRQECLDD	1.36%
	p.G12R	DRB1*11:04	181.75	VVVGARGVGKSALTI	0.68%
	p.G12S	DRB1*12:01	216.22	KLVVVGASGVGKSAL	1.36%
	p.G12Y	DRB1*08:04	89.1	LVVVGAWRRQECLDD	0.68%
*MUC16*	p.A5415T	DRB1*08:03	491.36	TMHHSTNTAVTNVGT	0.68%
	p.D1142Y	DRB1*01:02	467.11	PYPGSARSTWLGILS	0.68%
	p.D9418Y	DRB1*01:02	42.61	SRGPEYVSWPSPLSV	0.68%
	p.E11272V	DRB1*12:01	72.72	ISLVTHPAVSSSTLP	0.68%
	p.E14134Q	DRB1*08:03	201.38	QLISLRPQKDGAATG	0.68%
	p.E8581D	DRB1*04:04	399.26	FFSTLPDSISSSPHP	0.68%
	p.G13025 V	DRB1*01:02	161.1	TNLQYGGHASPWLQE	0.68%
	p.G13669C	DRB1*04:05	479.01	KFNTTERVLQCLLRS	0.68%
	p.G1530 V	DRB1*12:01	162.76	GIRSLGRTVDLTTVP	0.68%
	p.G3326R	DRB1*01:02	379.94	VSLESPTARSITRTG	0.68%
	p.G6740C	DRB1*04:01	419.34	TIITRTCPPLGSTSQ	0.68%
	p.H12349N	DRB1*04:04	254.12	NSLYVNGFTNQSSVS	0.68%
	p.H14021N	DRB1*01:03	422.78	HELSQQTNGITRLGP	0.68%
	p.L12891I	DRB1*08:03	420.3	LQGLIGPMFKNTSVG	0.68%
	p.L2407I	DRB1*11:04	376.36	SSSPSIFSSDRPQVP	0.68%
	p.L8172I	DRB1*04:03	461.25	GFAQITVSPETSTET	0.68%
	p.M3792 T	DRB1*04:01	445.39	ITSAVTPAATARSSG	0.68%
	p.N787Y	DRB1*12:01	57.97	ATSPERVRYATSPLT	0.68%
	p.P1203A	DRB1*01:02	129.39	TTSLTASNIPTSGAI	0.68%
	p.P12152H	DRB1*03:01	473.39	RPDHEDLGLDRERLY	0.68%
	p.P242H	DRB1*12:01	266.64	YSSFLDLSHKGTPNS	0.68%
	p.P2978fs	DRB1*01:02	395.63	VPLQEQGTLDMPQRA	0.68%
	p.P841L	DRB1*12:01	26.68	STLSLLSVSGVKTTF	0.68%
	p.P8502A	DRB1*11:03	392.32	AESAITIETGSAGAT	0.68%
	p.S13403I	DRB1*12:01	253.18	DPKIPGLDRERLYWK	0.68%
	p.S1887C	DRB1*04:01	315.03	KSLCMGNSTHTSMTY	0.68%
	p.S3428Y	DRB1*04:01	396.78	TSYWSDQTSGSDITL	0.68%
	p.S490Y	DRB1*01:01	88.64	TTGSTYGRQSSSTAA	0.68%
	p.S586Y	DRB1*01:02	479	TYADTLIGESTAGPT	0.68%
	p.S6935F	DRB1*11:04	66.51	TSMSVFSETTKIKRE	0.68%
	p.S7304Y	DRB1*16:02	107.01	MLPEIYTTRKIIKFP	0.68%
	p.S8560C	DRB1*13:03	414.19	VEEASCVSSSLSSPA	0.68%
	p.T12805S	DRB1*11:03	420.91	NGIKELGPYSLDRNS	0.68%
	p.T435 K	DRB1*13:03	301.6	EGTLNKSMTPLETSA	0.68%
	p.T7989R	DRB1*13:03	492.12	SRLPESISSSPLPVT	0.68%
	p.T8159A	DRB1*01:02	487.68	VSRTEVASSSRTSIS	0.68%
	p.V11743 M	DRB1*01:02	292.47	SPGAPEMMTSQITSS	0.68%
*TP53*	p.A161V	DRB1*13:02	344.62	RVRAMVIYKQSQHMT	0.68%
	p.A69fs	DRB1*08:03	238.65	QLRFPSGLLAFWDSQ	0.68%
	p.C135F	DRB1*04:07	85.99	LNKMFFQLAKTCPVQ	0.68%
	p.C176F	DRB1*12:01	140.21	YKQSQHMTEVVRRFP	0.68%
	p.C176Y	DRB1*12:01	163.66	YKQSQHMTEVVRRYP	0.68%
	p.C277F	DRB1*11:04	200.58	VRVCAFPGRDRRTEE	1.36%
	p.D281E	DRB1*11:04	412.76	VRVCACPGRERRTEE	0.68%
	p.D281Y	DRB1*01:02	286.24	RVCACPGRYRRTEEE	0.68%
	p.E271K	DRB1*11:04	283.03	GRNSFKVRVCACPGR	0.68%
	p.E285K	DRB1*11:04	418.37	VRVCACPGRDRRTKE	0.68%
	p.F270 V	DRB1*13:03	401.66	NLLGRNSVEVRVCAC	0.68%
	p.G245C	DRB1*08:04	255.62	NSSCMGCMNRRPILT	0.68%
	p.G334 V	DRB1*03:01	268.4	DGEYFTLQIRVRERF	0.68%
	p.M237I	DRB1*07:01	251.46	DCTTIHYNYICNSSC	0.68%
	p.N239S	DRB1*07:01	246.03	YNYMCSSSCMGGMNR	0.68%
	p.P152fs	DRB1*01:01	230.65	PVQLWVDSTPRPAPA	0.68%
	p.P278H	DRB1*11:04	318.15	VRVCACHGRDRRTEE	0.68%
	p.R158L	DRB1*12:01	329.17	STPPPGTRVLAMAIY	0.68%
	p.R175H	DRB1*01:02	468.06	MTEVVRHCPHHERCS	1.36%
	p.R273C	DRB1*01:02	494.42	EVCVCACPGRDRRTE	0.68%
	p.R280I	DRB1*01:02	445.57	VRVCACPGIDRRTEE	0.68%
	p.R337L	DRB1*01:01	225.82	FTLQIRGRELFEMFR	0.68%
	p.S127C	DRB1*16:01	380.13	VTCTYCPALNKMFCQ	0.68%
	p.V73 fs	DRB1*01:02	15.9	WPLHQQLLHRRPLHQ	0.68%
*EGFR*	p.709_710ET > D	DRB1*16:02	411.98	SGEAPNQALLRILKE	1.36%
	p.773_774insH	DRB1*12:01	281.83	VMASVDNPHVCRLLG	0.68%
	p.ELR746del	DRB1*01:02	169.33	ELREATSPKANKEIL	1.36%
	p.ELREA746del	DRB1*01:02	77.85	KELREATSPKANKEI	0.68%
	p.K754I	DRB1*01:03	230.19	ELREATSPIANKEIL	0.68%
	p.L858R	DRB1*08:03	205.08	ITDFGRAKLLGAEEK	1.36%
	p.L861Q	DRB1*09:01	464.7	TDFGLAKQLGAEEKE	0.68%
	p.Q432H	DRB1*16:01	363.6	LEIIRGRTKHHGQFS	0.68%
	p.S768I	DRB1*03:01	212.84	AYVMAIVDNPHVCRL	0.68%
	p.TSPKANKE751del	DRB1*01:01	95.63	IKELREATSPKANKE	0.68%
	p.V769 L	DRB1*04:04	253.85	VMASLDNPHVCRLLG	0.68%

## Discussion

Several groups have proposed to predict HLA Class II presented neo-antigens through next generation sequencing for cancer immunotherapy [1–6]. In both mouse models and human patients, the function of predicted neo-antigens have been verified,by measuring CD4 T cell responses or tumor rejection.

In this study, we have predicted the HLA Class II-presented neo-antigen peptides in lung adenocarcinoma. An average of 59 HLA DRB1-presented neo-antigen mutations were predicted per lung cancer patient. This prediction is based on the assumption that all HLA DRB1 alleles may be the MHC class II molecule to present mutated peptides in a patient. Since a specific cancer patient only express one HLA DRB1 allele, the actual mutant peptide epitope presented by a cancer patient is much lower. Unfortunately, the HLA DRB1 allele data are not available in public TCGA database for the lung cancer patients we have studied. Assuming HLA DRB1*01:01 is the HLA DRB1 allele, 54 strong binders and 896 weak binders were found in 147 patients. In average, 5 mutant peptides were found per patient with HLA DRB1*01:01 allele.

van Buuren et al. reported that the sensitivity of neo-epitope prediction from analysis of exonic SNVs in cancer exome sequencing data requires little improvement [20]. Our analysis on mutant peptides presented by HLA Class I molecules in lung cancer patients is consistent with this conclusion (Additional file 1: Table S1 and Additional file 5: Table S5, top mutated genes with predicted epitopes binding to HLA Class I molecules).

A weakness of our analysis is that the expression of predicted neo-epitopes could not be determined. As we described, genes with 3 or more reads covered in RNA-seq data were defined as expressed according to Kandoth et al. [12]. Although the normal copy of a gene may be expressed, its variants may not be expressed, especially truncating variants that may undergo nonsense-mediated transcript decay. Mass spectrometry-based new technologies are emerging to verify predicted neo-epitopes [21–23], through analysis of eluted peptides from HLA molecules purified from cancer tissues.

K-Ras, TP53, and EGFR mutants are well known vaccine candidates which are currently in clinical trials [24–27]. Our data suggest that such mutations in proliferation-related genes are also candidate for CD4 epitopes. In addition, neo-antigens of passenger mutations are also attractive targets for individualized precision therapy. There is urgent need for technologies which may help to determine whether the predicted neo-antigen mutations are presented by HLA Class II molecules. Technical platforms include ELISPOT assay by synthetic candidate peptide epitopes, T cell stimulation assay by using antigen presenting cell lines expressing specific HLA DRB1 molecules, and tetramer staining-based sorting of neoantigen-specific T cells.

## Conclusions

This study used clonal mutation analysis to predict HLA DRB1 molecule presented neo-antigen mutant peptides which are expressed at RNA level. Genes discovered here provide clues for identifying CD4 T cell epitopes for immune monitoring and therapy.

## Additional files

Additional file 1: **Table S1.** Top mutated genes with predicted HLA Class I binding neo-peptides in 147 lung adenocarcinoma patients in this study. T cell epitopes presented by MHC Class I molecules were predicted by NetMHCPan2.4 binding algorithm. (XLSX 192 kb)

Additional file 2: **Table S2.** Number of predicted neo-antigen peptides presented by MHC Class I molecules in 147 lung adenocarcinoma patients. T cell epitopes presented by MHC Class I molecules were predicted by NetMHCPan2.4 binding algorithm. MHC-I molecules which are significantly more frequent in presenting neo-antigens were labelled as bold according to *P* values. Significant levels were calculated using one sided Mann-Whitney U test. (XLSX 11 kb)

Additional file 3: **Table S3.** Amino acid sequences of predicted MHC class I binding neo-peptides of KRAS, EGFR, TP53, and MUC16 in 147 lung adenocarcinoma patients in this study. T cell epitopes presented by MHC Class I molecules were predicted by NetMHCPan2.4 binding algorithm. (XLSX 13 kb)

Additional file 4: **Table S4.** Amino acid sequences of predicted MHC Class II molecule HLA DRB1 binding neo-peptides in 147 lung adenocarcinoma patients in this study. (XLSX 503 kb)

Additional file 5: **Table S5.** Amino acid sequences of predicted MHC class I binding neo-peptides in 147 lung adenocarcinoma patients in this study. (XLSX 534 kb)

## Abbreviations

CCF: Cancer cell fraction
EGFR: Epidermal growth factor receptor
ELISPOT: Enzyme-Linked ImmunoSpot
HLA: Human leukocyte antigen
MHC: Major histocompatibility complex
SNP: Single nucleotide polymorphism
SNV: Single nucleotide variations
TCGA: The Cancer Genome Atlas
WES: Whole exon sequencing
